# A decrease in plant gain, namely CO_2_ stores, characterizes dysfunctional breathing whatever its subtype in children

**DOI:** 10.3389/fphys.2023.1217391

**Published:** 2023-07-04

**Authors:** Plamen Bokov, Claudine Peiffer, Jorge Gallego, Jade Pautrat, Boris Matrot, Christophe Delclaux

**Affiliations:** ^1^ Service de Physiologie Pédiatrique -Centre du Sommeil—CRMR Hypoventilations Alvéolaires Rares, AP-HP, Hôpital Robert Debré, INSERM NeuroDiderot, Université de Paris, Paris, France; ^2^ Service de Physiologie Pédiatrique, Assistance Publique - Hôpitaux de Paris (AP-HP), Hôpital Robert Debré, Paris, France; ^3^ INSERM NeuroDiderot, Université de Paris, Paris, France

**Keywords:** hyperventilation, loop gain, controller gain, hyperventilation provocation test, dysfunctional breathing, ventilatory control

## Abstract

**Background:** Whether dysfunctional breathing (DB) subtype classification is useful remains undetermined. The hyperventilation provocation test (HVPT) is used to diagnose DB. This test begins with a 3-min phase of hyperventilation during which fractional end-tidal CO_2_ (FETCO_2_) decreases that could be an assessment of plant gain, which relies on CO_2_ stores. Our aim was to assess 1) whether the children suffering from different subtypes of DB exhibit decreased plant gain and 2) the relationships between HVPT characteristics and plant gain.

**Methods:** We retrospectively selected 48 children (median age 13.5 years, 36 females, 12 males) who exhibited during a cardiopulmonary exercise test either alveolar hyperventilation (transcutaneous PCO_2_ < 30 mmHg, *n* = 6) or inappropriate hyperventilation (increased VE’/V’CO_2_ slope) without hypocapnia (*n* = 18) or dyspnea without hyperventilation (*n* = 18) compared to children exhibiting physiological breathlessness (dyspnea for sports only, *n* = 6). These children underwent tidal-breathing recording (ventilation and FETCO_2_ allowing the calculation of plant gain) and a HVPT.

**Results:** The plant gain was significantly higher in the physiological group as compared to the dyspnea without hyperventilation group, *p* = 0.024 and hyperventilation without hypocapnia group, *p* = 0.008 (trend for the hyperventilation with hypocapnia group, *p* = 0.078). The slope of linear decrease in FETCO_2_ during hyperventilation was significantly more negative in physiological breathlessness group as compared to hyperventilation without hypocapnia group (*p* = 0.005) and dyspnea without hyperventilation group (*p* = 0.049).

**Conclusion:** The children with DB, regardless of their subtype, deplete their CO_2_ stores (decreased plant gain), which may be due to intermittent alveolar hyperventilation, suggesting the futility of our subtype classification.

## 1 Introduction

The term “dysfunctional breathing” (DB) refers to a set of different types of changes in breathing patterns associated with a variety of acute or chronic symptoms that may be respiratory, notably dyspnea, and/or non-respiratory in the absence or in excess of organic disease, affecting both adults and children (Boulding et al., 2016). Several classification systems reflecting essentially expert opinions have been proposed (Boulding et al., 2016; Barker et al., 2020). Among these classification systems, hyperventilation syndrome (HVS) is the most popular and the earliest described form (Boulding et al., 2016). Therefore, the most commonly used supposedly diagnostic tests for HVS and even DB—i.e., the hyperventilation provocation test (HVPT) and the Nijmegen questionnaire—were initially focused on the detection of hyperventilation and what has been considered a mandatory consequence (i.e., hypocapnia and its related symptoms). Subsequently, however, the relative contribution of hyperventilation and hypocapnia to HVS and DB as well as their relationship with the corresponding symptoms were far more complex. Indeed, as first shown by Hornsveld et al. (1996), during daily life, the occurrence of symptoms is not systematically associated with hypocapnia, and the latter can be induced by isocapnic hyperventilation. Furthermore, as opposed to adults, in children, despite typical symptoms of HVS or DB, hypocapnia is generally lacking during both quiet breathing and the cardiopulmonary exercise test (CPET) (Mahut et al., 2014; Peiffer et al., 2022).

More recently, several CPET studies have identified exercise-induced inappropriate hyperventilation, as documented by an increase in the V’E/V’CO2 slope without overt hypocapnia in a subgroup of subjects suspected of having HVS or DB or in those with unexplained exertional dyspnea after a COVID-19 infection (Ionescu et al., 2020; Frésard et al., 2022). In these subjects, hyperventilation was generally associated with a rapid shallow breathing pattern and/or an inappropriately rapid increase in breathing frequency during exercise (Ionescu et al., 2020; Frésard et al., 2022; Peiffer et al., 2022). Thus, once again, the determination of DB subtypes seems not to be justified.

DB may also be associated with abnormal respiratory control. In a previous study, (Bokov et al., 2016), we assessed ventilatory control in adult patients with HVS, which included the calculation of loop gain, which evaluates the overall stability of the feedback system controlling ventilation, as well as of its two main components—i.e., controller gain, which is the change in ventilation per change in unit PaCO_2_ (determined by chemical drive), and the plant gain, reflecting the change in PaCO_2_ per unit change in ventilation (reflecting the effectiveness of the lung to modify blood gas). Our results showed that compared to the healthy control subjects, plant gain was significantly lower in patients with intermittent hypocapnia (only during exercise) and even lower in patients with chronic hyperventilation (Bokov et al., 2016). Modelling studies have shown that plant gain depends on PvCO_2_ (i.e., on CO_2_ tissue stores) (Carley and Shannon, 1988). This means that patients with intermittent alveolar hyperventilation deplete, to some extent, their CO_2_ stores, which is reflected by decreased plant gain. Thus, a decrease in plant gain may traduce intermittent hypocapnia.

The primary objective of our retrospective study was to assess whether or not children suffering from different subtypes of DB exhibit decreased plant gain. We hypothesized that all children with DB, whether or not hypocapnia was evidenced during a CPET, may exhibit similar kinetics of PETCO_2_ decrease during the HVPT and similar levels of plant gain measured during tidal ventilation. The secondary objective was to assess the relationship between plant gain and the HVPT. This test begins with a 3 min phase of hyperventilation during which PETCO_2_ is recorded (Pauwen et al., 2022). The PETCO_2_ decrease in response to augmented ventilation could be an assessment of plant gain. The ratio ΔPACO_2_/ΔV’E reflects how efficiently the lungs excrete CO_2_ and is known as plant gain.

## 2 Materials and methods

### 2.1 Participants

**FIGURE 1:**
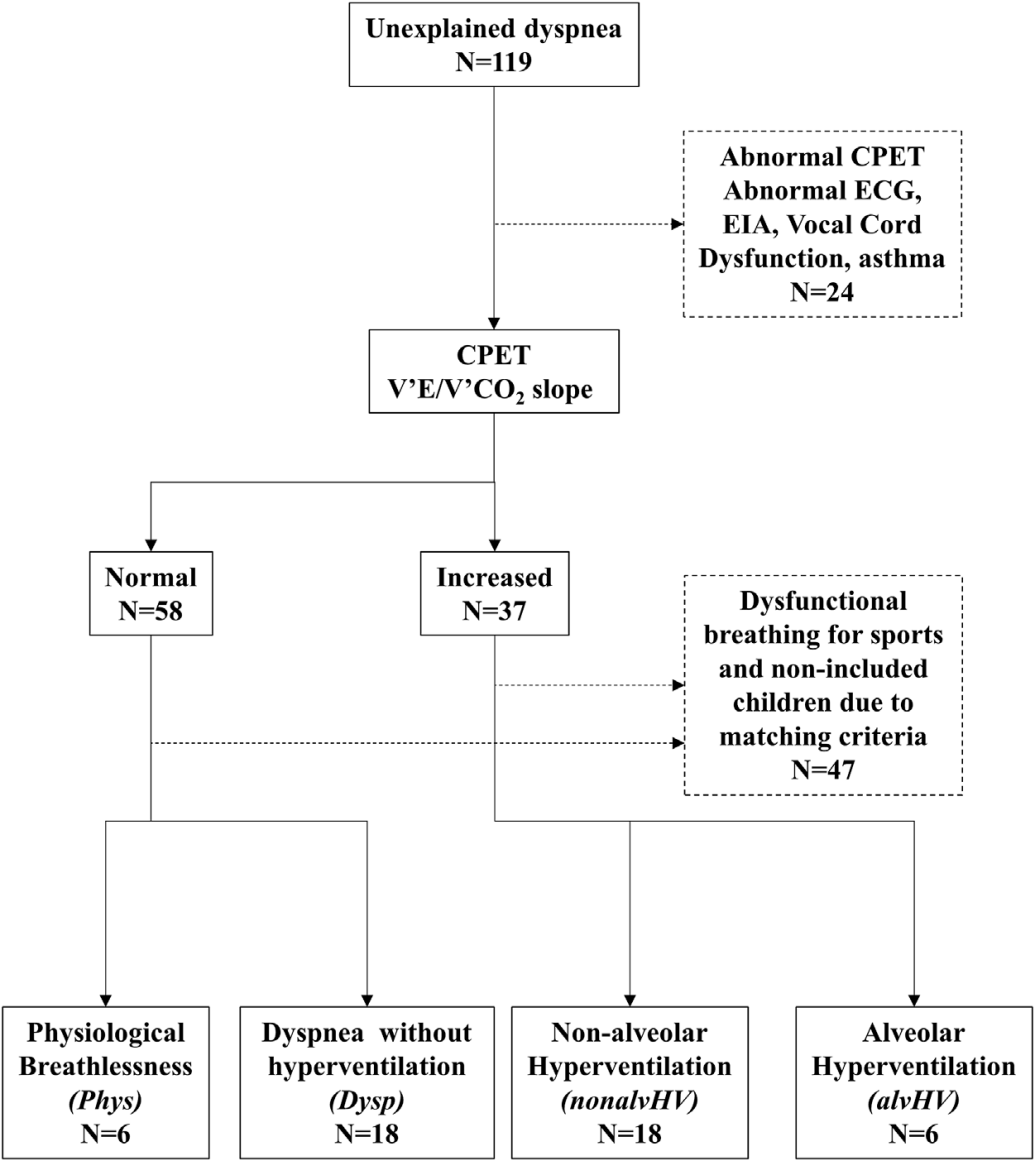
Selection process of the 48 children.

The participants of our study were selected from our database that collects all patients referred to our clinic for unexplained or disproportional dyspnea since March 2018. One hundred and nineteen children were included. Four groups of children were selected: 1) children with physiological dyspnea on exercise (normal lung function, normal echocardiography, dyspnea on exercise for sports without resting dyspnea, normal VE’/V’CO2 slope during CPET, and Nijmegen score <23: Phys group), 2) children with DB and alveolar hyperventilation on exercise (increased VE’/V’CO_2_ slope with hypocapnia, either permanent or at the recovery of exercise defined by transcutaneous PaCO_2_ < 30 mmHg: alvHV group), 3) children with DB with hyperventilation on exercise without hypocapnia (increased VE’/V’CO_2_ slope on exercise with PaCO_2_ ≥ 30 mmHg: nonalvHV group), and 4) children with DB without hyperventilation on exercise (dyspnea on exercise on daily activities such as walking and normal VE’/V’CO_2_ slope on exercise: Dysp [Dyspnea] group). The level of PaCO_2_ used to define hypocapnia in children is justified by the results of our previous study (Mahut et al., 2014). Thus, while the level of exercise that induces dyspnea differentiates the Phys and Dysp groups, in the latter group, resting dyspnea could also be present. All children with Dysp had an absence of overt current disease, notably respiratory and/or cardiopulmonary, based on normal or near normal CPET (notably without any evidence of ECG abnormality, inspiratory airflow limitation, or post-exercise bronchoconstriction), lung function, chest radiography, and echocardiography (these children were recruited prior to the COVID-19 pandemic). We selected similar numbers of children for both the Phys/alvHV groups and the nonalvHV/Dysp groups with non-significant differences for both age and sex since these factors may affect the results of the HVPT (Peiffer et al., 2022). The selection process of the 48 children is described in Figure 1.

### 2.2 Cardiopulmonary exercise test

**FIGURE 2:**
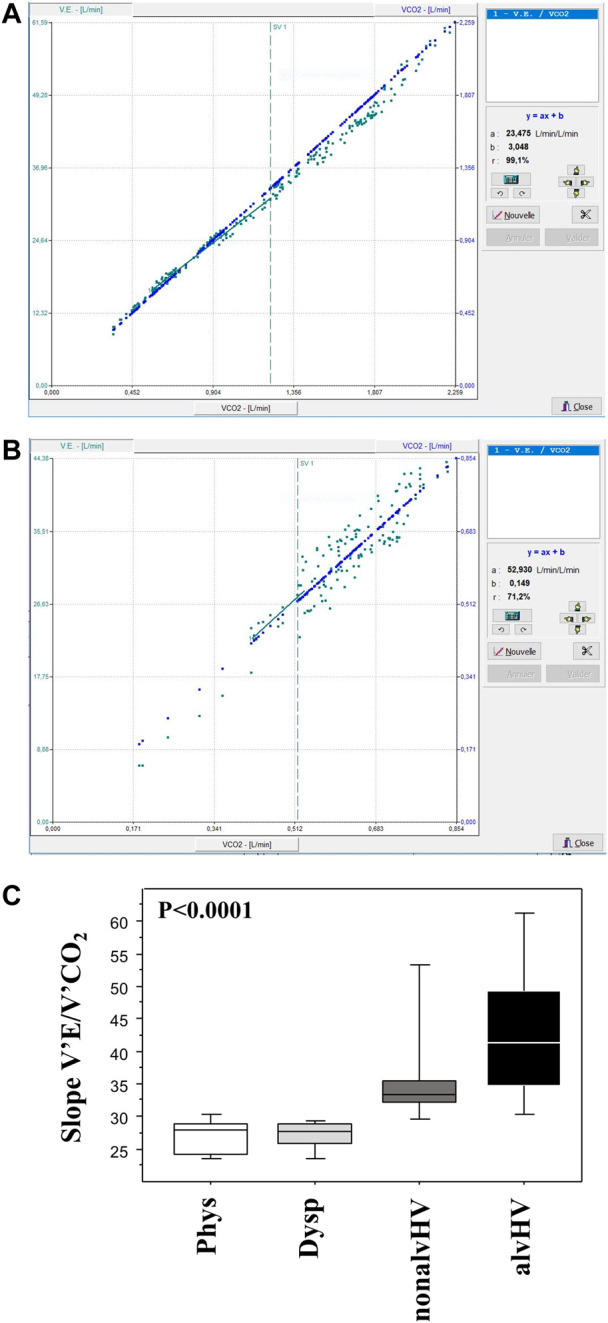
Calculation of the V’E/V’CO_2_ slopes. The slope of V’E/V’CO_2_ was calculated from 1 min of exercise to the anaerobic threshold (SV1) as illustrated in a child with a normal slope (panel A) and in a child with an increased slope (panel B, also showing variable V’E after ventilatory threshold). Panel C summarizes the data of these slopes of the four groups of children: Phys (*n* = 6) is the physiological breathlessness group, Dysp (*n* = 18) is the group with dyspnea (without hyperventilation on exercise), nonalvHV (*n* = 18) is the group with hyperventilation on exercise without hypocapnia and alvHV (*n* = 6) is the group with alveolar hyperventilation (hypocapnia) on exercise. The *p* value of the intergroup comparison is given but the high degree of significance is related to the selection of groups based on the slope calculation.

CPETs were performed as previously described (Mahut et al., 2014; Peiffer et al., 2022). Briefly, all the tests consisted of a period of 3 min of rest (after baseline spirometry), 2 min of warm-up (20–30 W), the incremental work rate period (rate of 5–20 W min judged by the same operator), and a 10 min resting recovery period. The subjects were asked to score their breathlessness using the Borg scale at the baseline, 30% and 40% of maximal ventilation, and at peak exercise. Ventilation was expressed as the percentage of predicted maximal voluntary ventilation (calculated as 35 × FEV_1_). Spirometry was performed at the baseline and 3, 6, and 10 min after the end of exercise. The decrease in FEV_1_ was calculated as a percentage of baseline value. The slope of V’E/V’CO_2_ was calculated from 1 min of exercise to the anaerobic threshold according to Cooper et al. (1987) and is illustrated in Figure 2. The reference equations of CPET parameters were those of Cooper et al. (1984).

### 2.3 Hyperventilation provocation test

The HVPT included the recording of 3 min of resting breathing, 3 min of voluntary hyperventilation, and 5 min of recovery, as previously described (Pauwen et al., 2022; Peiffer et al., 2022).

The following parameters were obtained from the recordings of each participant: mean and maximum values of FETCO_2_ of resting breathing, minimum value of voluntary hyperventilation, and kinetics of FETCO_2_ decrease during hyperventilation and FETCO_2_ increase during recovery. These two phases were fitted with a linear relationship: FETCO_2_ = slope × time + intercept, as done by other researchers (Han et al., 1997). The *r* ^2^ value of the linear regression was also recorded. Based on the individual linear regressions, the FETCO_2_ values at 3 min and 5 min of recovery were calculated. We also calculated a 3 min ratio (highest baseline FETCO_2_ over FETCO_2_ after 3 min recovery) and a 5 min ratio (highest baseline FETCO_2_ over FETCO_2_ after 5 min recovery) as done by other researchers (Vansteenkiste et al., 1991a). The hyperventilation phase and the recovery phase were also fitted with exponential and logarithmic models, respectively, as previously done (Peiffer et al., 2022).

### 2.4 Tidal breathing measurements and loop gain model

Recordings of tidal breathing were performed as previously described, (Bokov et al., 2016; Bokov et al., 2020), lasting 15 min, with the first 5 min being discarded.

The model was fitted on the changes from the baseline (mean) levels of the ventilatory parameters (V’E [minute ventilation] and PETCO_2_) that were obtained from tidal breathing measurements. We considered three distinct frequency bands: low-frequency oscillations with periods of 16–50 breaths/cycle, medium-frequency oscillations of 5–15 breaths/cycle, and high-frequency oscillations of 2–4 breaths/cycle. The means of the absolute values of the corresponding variables were given over the frequency ranges for loop gain and over all frequencies for plant and controller gains since we did not expect cyclical modifications of ventilation in DB (Bokov et al., 2016). On the other hand, a decrease in plant gain could lead to decreased loop gain (assessment of ventilator stability) over specific frequencies, which justifies the three frequency bands for loop gain. We used one constrained bivariate (V’E and PETCO_2_) model, as previously described (Bokov et al., 2018).

### 2.5 Questionnaires

The Nijmegen questionnaire, a measure of functional respiratory complaints that has not been validated in children, (Dixhoorn and Duivenvoorden, 1985; Dixhoorn and Folgering, 2015), but that has already been used in children to diagnose dysfunctional breathing (Groot et al., 2013) was considered as abnormal when ≥23 (Dixhoorn and Folgering, 2015). We previously evidenced a moderate correlation between this questionnaire and another questionnaire specifically devoted to children (Peiffer et al., 2022). Anxiety was evaluated using the State-Trait Anxiety Inventory for Children (STAI-C), as previously done (Mahut et al., 2014).

### 2.6 Statistical analysis

In our previous study in adults, (Bokov et al., 2016), we demonstrated a decrease in plant gain in patients with chronic hyperventilation (*n* = 6) compared to those with exercise-induced hyperventilation (*n* = 17). Consequently, the sample size of the two restricted groups (Phys and avlHV) was arbitrarily set at 6 and the sample size of the two other groups (nonalvHV and Dysp) at 18. Based on the slope of FETCO_2_ decrease obtained in our previous study in children with Dysp (−0.0065 ± 0.0020), we calculated that a sample size of 6 patients in the alvHV group or 18 in the nonalvHV and Dysp groups versus 6 patients in the Phys group allowed to demonstrate a significant difference (α 5%, β 80%), with the Phys group having a slope of −0.0097 or −0.0092, respectively.

The results are expressed as median [25^th^–75^th^ percentile] for continuous data and as frequency (percentage) for categorical data. The comparisons among the four groups of children were made using the Kruskal–Wallis test for continuous variables. Subgroup comparisons were made using the Mann–Whitney test. Comparisons of the categorical variables were made using the chi-squared test (or Fisher test when necessary). Linear correlations were evaluated using the Pearson test. A *p* value < 0.05 was deemed significant. No correction for multiple testing was done given the pathophysiological design of the study (Rothman, 1990). Statistical analyses were performed with the StatView 5.0 (SAS institute, Cary, NC) software.

## 3 Results

The clinical and CPET characteristics of the four groups of children are described in Table 1. Given the selection criterion, the Phys group had no resting dyspnea, whereas 37 out of 42 (88%, 95% confidence interval: 74%–96%) children with DB, whatever the subtype, had resting dyspnea.

**TABLE 1:** Description of the four groups of children: clinical and CPET descriptors.

	Phys group	alvHV group	nonalvHV group	Dysp group	*p* value
	N = 6	N = 6	N = 18	N = 18	
Age, years	13.0 [10.9; 13.6]	11.4 [9.3; 15.6]	13.4 [10.5; 15.6]	14.4 [11.1; 15.6]	0.511
Sex*, Female/Male, n	4/2	5/1	12/6	15/3	0.614
Height, cm	155 [144; 162]	143 [134; 164]	158 [146; 174]	155 [147; 160]	0.444
Weight, kg	49 [39; 57]	35 [28; 55]	47 [39; 61]	48 [38; 56]	0.353
Body mass index, kg.m^-2^	21.2 [18.8; 22.0]	18.2 [16.2; 20.4]	18.7 [18.1; 22.7]	20.0 [17.4; 21.8]	0.461
Resting dyspnea, n	0	6	15	16	ND
Nijmegen score	14 [12; 15]	16 [9; 22]	20 [18; 24]	26 [18; 29]	ND
STAI-C score	24 [19; 29]	39 [34; 41]	37 [34; 40]	34 [31; 43]	**0.029**
CPET results					
Dyspnea Borg score at rest	0 [0; 0]	0 [0; 0]	0 [0; 0]	0 [0; 0]	0.937
Borg score 30% V’E max pred	0.5 [0; 2]	1 [0.5; 3]	3 [1; 3]	2 [1; 3]	0.244
Borg score 40% V’E max pred	0.5 [0.5; 2]	3 [0; 5]	5 [3; 7]	3 [2; 5]	**0.010**
Peak Borg score	7 [5; 9]	7 [1; 7]	7 [5; 9]	7 [5; 9]	0.545
Symptoms, stop effort					
Dyspnea/leg fatigue/both, n	0/1/5	1/2/3	2/6/10	1/6/11	ND
Dyspnea descriptors					
- effort, n	5	3	8	10	
- air hunger, n	1	3	6	10	
- chest tightness, n	0	1	5	2	
- undetermined, n	1	0	2	2	
Similar to usual complaint, n^a^	3	6	14	12	ND
Slope V’E/V’CO_2_	27.9 [24.2; 28.8]	41.3 [34.8; 49.2]	33.4 [32.2; 35.5]	27.7 [25.8; 28.9]	ND
Nadir PCO_2_, mmHg	32.3 [31.6; 33.2]	28.1 [23.2; 29.0]	32.7 [32.0; 34.5]	32.5 [31.3; 34.0]	ND
HR peak, % predicted	89 [82; 98]	92 [80; 97]	89 [80; 93]	90 [85; 97]	0.946
QR peak	1.03 [1.02; 1.16]	1.13 [0.99; 1.27]	1.15 [1.04; 1.20]	1.16 [1.09; 1.25]	0.712
V’O_2_ peak, % predicted	77 [70; 99]	67 [51; 75]	68 [57; 78]	73 [63; 91]	0.332
Watts peak, % predicted	71 [61; 77]	60 [49; 63]	62 [52; 67]	64 [55; 70]	0.236
O_2_ pulse peak, % predicted	95 [70; 115]	76 [55; 81]	75 [66; 91]	80 [77; 99]	0.323
Ventilatory threshold, %V’O_2_max pr	40 [34; 64]	39 [32; 44]	42 [32; 47]	41 [36; 51]	0.826
Ventilatory reserve peak, %	33 [25; 44]	39 [32; 46]	40 [35; 50]	40 [36; 46]	0.926

ND, not done due to selection criteria; *: sex assigned at birthx.

Statistically significant results are highlighted in bold.

^a^ At the end of the CPET, it was asked to the children whether the test reproduced the usual exercise symptoms.

The only significant differences for the four groups of children concerning clinical and CPET descriptors were a lower STAI-C score in the Phys group compared to the three other groups (versus alvHV, *p* = 0.028; versus nonalvHV, *p* = 0.006; versus Dysp, *p* = 0.006) and lower dyspnea on exercise at 40% of the predicted maximal minute ventilation compared to two other groups (versus nonalvHV, *p* = 0.003; versus Dysp, *p* = 0.009). There was no significant difference for dyspnea at 40% of the predicted maximal minute ventilation versus the alvHV group (*p* = 0.456).

**FIGURE 3:**
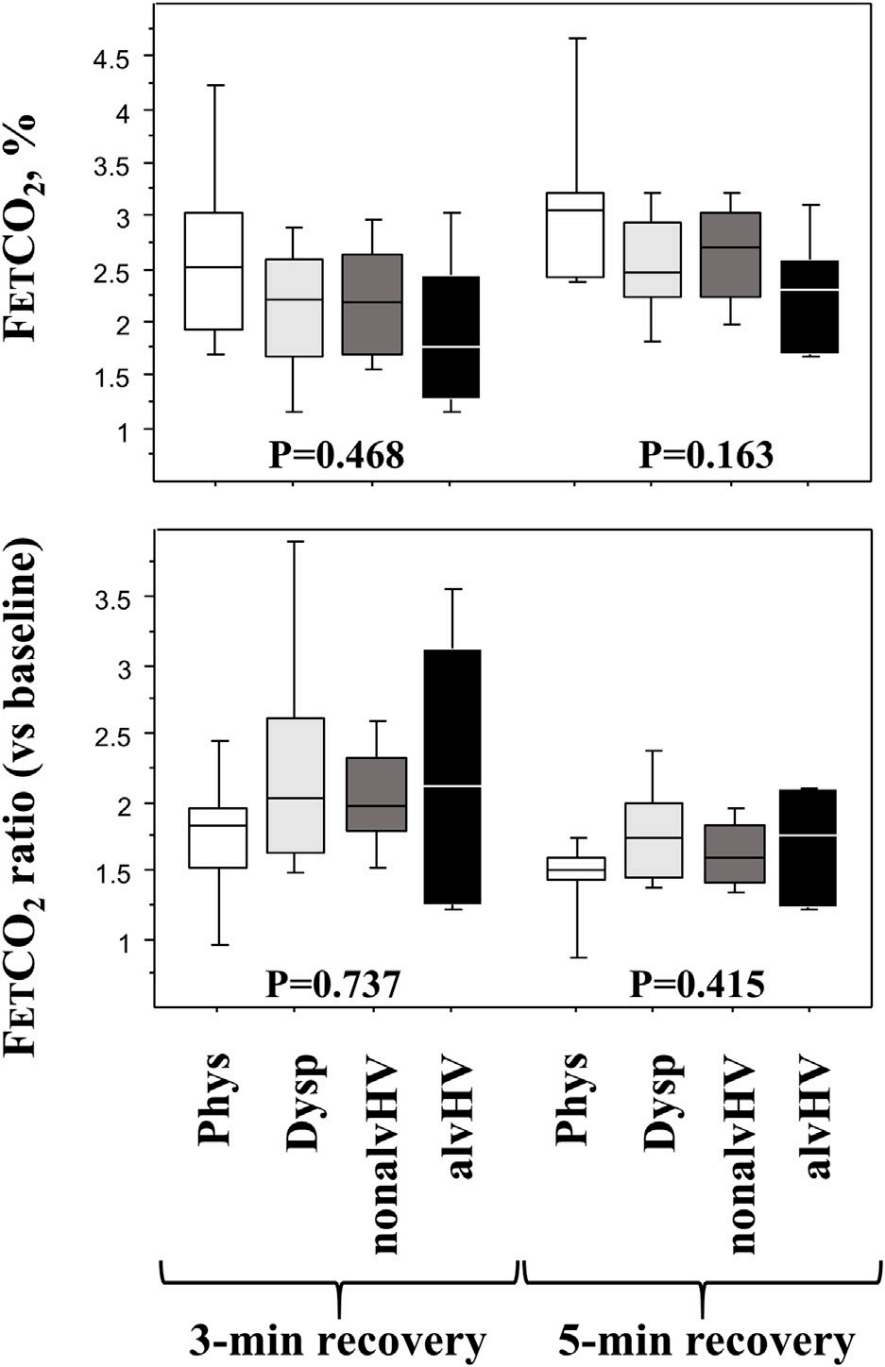
Diagnosis parameters of the HPVT at the recovery phase. Upper panels: FETCO_2_ at 3 min (left panel) and 5 min (right panel) of recovery (after 3-min hyperventilation) are described in the four groups of children: Phys (*n* = 6) is the physiological breathlessness group, Dysp (*n* = 18) is the group with dyspnea (without hyperventilation on exercise), nonalvHV (*n* = 18) is the group with hyperventilation on exercise without hypocapnia and alvHV (*n* = 6) is the group with alveolar hyperventilation (hypocapnia) on exercise. Lower panels: 3-min ratio (left panel: highest base line FETCO_2_ over FETCO_2_ after 3 min recovery) and a 5-min ratio (right panel: highest base line FETCO_2_ over FETCO_2_ after 5 min recovery) in the four groups of children. *p* values are Kruskall Wallis tests.

The results of the loop gain parameters are described in Table 2, while the results of the HVPT are described in Table 2 and Figure 3.

**TABLE 2:** Results of the HVPT and loop gain.

	Phys group	alvHV group	nonalvHV group	Dysp group	*p* value
	N = 6	N = 6	N = 18	N = 18	
HVPT parameters					
Baseline mean FETCO_2_, %	4.76 [4.43; 4.89]	3.88 [3.36; 4.60]	4.55 [4.08; 4.83]	4.45 [3.99; 4.71]	0.281
*FETCO*_*2*_ *linear decrease* (*HV*)					
Slope, %.s^-1^	−0.0083 [-0.0092; −0.0076]	−0.0037 [-0.0086; −0.0025]	−0.0054 [-0.0064; −0.0040]	−0.0074 [-0.0083; −0.0045]	**0.031**
Intercept, %	5.83 [5.74; 5.98]	4.81 [3.70; 5.42]	4.52 [4.06; 5.32]	4.92 [4.13; 5.28]	**0.005**
*r* ^2^ of the linear decrease	0.82 [0.77; 0.85]	0.56 [0.32; 0.72]	0.69 [0.55; 0.81]	0.65 [0.38; 0.85]	0.209
Minimal FETCO_2_ during HV, %	2.17 [1.65; 2.60]	2.32 [2.12; 2.67]	2.54 [2.20; 2.76]	2.12 [1.98; 2.52]	0.169
*FETCO*_*2*_ *increase* (*recovery*)					
slope, %.s^-1^	0.0046 [0.0041; 0.0050]	0.0031 [0.0005; 0.0046]	0.0046 [0.0026; 0.0056]	0.0035 [0.0019; 0.0051]	0.257
intercept, %	1.70 [1.25; 2.75]	1.20 [0.31; 2.45]	1.34 [0.95; 2.08]	1.50 [0.87; 2.21]	0.780
r^2^ of the linear increase	0.52 [0.47; 0.61]	0.37 [0.20; 0.45]	0.46; 0.20; 0.75]	0.45 [0.20; 0.61]	0.549
Loop Gain parameters					
Plant gain_AF_, mmHg.min.L^-1^	0.36 [0.30; 0.40]	0.15 [0.14; 0.17]	0.10 [0.06; 0.16]	0.14 [0.09; 0.21]	**0.033**
Controller gain_AF_, L.mmHg.min^-1^	0.15 [0.09; 0.21]	0.10 [0.06; 0.24]	0.09 [0.04; 0.15]	0.11 [0.04; 0.16]	0.434
Loop gain-Low frequency	0.405 [0.108; 0.500]	0.194 [0.170; 0.200]	0.182 [0.051; 0.347]	0.202 [0.092; 0.317]	0.655
Loop gain-Medium frequency	0.050 [0.047; 0.068]	0.024 [0.020; 0.030]	0.018 [0.007; 0.038]	0.034 [0.009; 0.054]	0.262
Loop gain-High frequency	0.011 [0.010; 0.013]	0.005 [0.04; 0.007]	0.002 [0.001; 0.005]	0.006 [0.001; 0.011]	**0.003**

Plant gain_AF_, denotes plant gain measured over all frequencies (see methods).

Statistically significant results are highlighted in bold.

Both plant gain and loop gain at high frequencies were significantly different among the four groups. Plant gain was significantly higher in the Phys group compared to the Dysp group (*p* = 0.024) and nonalvHV group (*p* = 0.008), while a trend for higher plant gain was observed compared to the alvHV group (*p* = 0.078). Loop gain at high frequencies was significantly higher in the Phys group compared to the three other groups (Dysp group, *p* = 0.035; nonalvHV group, *p* < 0.001; alvHV group, *p* = 0.004).

The slope of linear decrease in FETCO_2_ during hyperventilation was significantly more negative in the Phys group compared to both the nonalvHV group (*p* = 0.005) and the Dysp DB group (*p* = 0.049). The intercept of this linear relationship was significantly higher in the Phys group compared to the three other groups (Dysp group, *p* < 0.001; nonalvHV group, *p* = 0.004; alvHV group, *p* = 0.004). The recovery criteria of FETCO_2_ are described in Figure 3, which shows a nonsignificant difference for any criterion at either 3 min or 5 min of recovery.

The kinetics of FETCO_2_ decay and subsequent recovery were calculated, giving a median decrease in the Phys group (*n* = 6) of −0.0083 × time (s) + 5.83 (median *r* ^2^ = 0.82) and a median increase of 0.0046 × time (s) + 1.70 (median *r* ^2^ = 0.52). The children with DB, whatever the subtype (*n* = 42), were characterized by a median decrease of −0.0055 × time (s) + 4.85 (median *r* ^2^ = 0.66) and a median increase of 0.0037 × time (s) + 1.44 (median *r* ^2^ = 0.45). The slopes and intercepts of the decrease were significantly different for these two groups (slope, *p* = 0.013; intercept, *p* < 0.001). The slopes and intercepts of the increase were not significantly different (0.676 and 0.355, respectively).

We further evaluated whether these kinetics of decrease and increase were better fitted by exponential and logarithmic functions, respectively. The median decrease in FETCO_2_ during hyperventilation in the Phys group was 7.16 e^−0.0025 × time^, and its median increase during recovery was 2.55 Ln(time)—11.83. The median *r* ^2^ values of these relationships were 0.83 and 0.55, respectively. The median decrease in FETCO_2_ during hyperventilation in the whole DB group was 5.66 e^−0.0020 × time^, and its median increase during recovery was 1.96 Ln(time)—8.55 (decrease: 7.16 and 2.55 were significantly different, *p* = 0.007). The median *r* ^2^ values of these relationships were 0.69 and 0.45, respectively. For simplicity, the other analyses were performed on the linear relationships.

### 3.1 Correlates of loop gain parameters

Plant gain correlated positively with the intercept of the FETCO_2_ recovery (*r* ^2^ = 0.13, *p* = 0.017), with the mean baseline FETCO_2_ (*r* ^2^ = 0.10, *p* = 0.043) and negatively correlated with the slope of FETCO_2_ recovery (*r* ^2^ = 0.10, *p* = 0.034). Controller gain did not correlate with any parameter of the HVPT. Loop gain (stability of ventilatory control) did no correlate with dyspnea.

### 3.2 Correlates of HVPT parameters

The slope of FETCO_2_ decreased linearly and correlated negatively with age (*r* ^2^ = 0.09, *p* = 0.043), the mean baseline FETCO_2_ (*r* ^2^ = 0.08, *p* = 0.044), and the slope of FETCO_2_ recovery (*r* ^2^ = 0.22, *p* < 0.001).

## 4 Discussion

The main results of this cross-sectional observational study demonstrate that a decrease in plant gain characterizes all patients with DB whatever their subtype—which may be due to intermittent alveolar hyperventilation, suggesting the futility of our subtype classification—and that the HVPT describes CO_2_ kinetics related at least partially to plant gain (CO_2_ stores).

First, the design of the study should be discussed. The sample sizes of both the physiological breathlessness and alveolar hyperventilation groups were restricted. We already showed that the latter group was restricted in children since only 3 out of 79 children with unexplained exertional dyspnea had frank alveolar hyperventilation during a CPET in a previous multicenter study (Mahut et al., 2014). The physiological breathlessness group may seem disputable, but based on our experience, some patients are referred because they want to know whether they experience dyspnea (abnormal process related to underlying disease) or normal breathlessness on exercise. For instance, children beginning endurance sport practice may ask this question, justifying our selection criterion. One may discuss whether this physiological breathlessness group was a healthy group given the *a posteriori* definition. Their median plant gain and loop gain were in the range of that previously evidenced in otherwise healthy children without obstructive sleep apnea, (Bokov et al., 2020), suggesting the absence of previous alveolar hyperventilation. Our study also has limitations given the restricted sample size of some groups, especially when significant differences were absent.

We previously hypothesized that dyspnea of breathing disorders may be related to central nervous system sensitization, (Bokov et al., 2016), which is an important concept for a therapeutic point of view (Dessel et al., 2014). Resting dyspnea could therefore be “allodyspnea” (dyspnea for a nondyspneic trigger such as sighing), whereas exercise-induced dyspnea may be “hyperdyspnea” (dyspnea that is disproportionate to the level of ventilation, as demonstrated by Jack et al.) (Jack et al., 2004). The results of this study confirm these statements since children with DB (all subtypes) had both allodyspnea (88%) and hyperdyspnea on exercise (increased Borg score at 40% V’E max predicted). Children with DB (all subtypes) were also more anxious (increased STAI-C score), which was expected given the correlation between the Nijmegen and STAI-C scores previously evidenced (Peiffer et al., 2022).

The main result of this study shows that plant gain and the kinetics of FETCO_2_ decrease during the HVPT are significantly different in children with physiological breathlessness compared to those with DB and that children with DB (only a trend for the group with alveolar hyperventilation) are characterized by decreased plant gain, whatever the DB subtype. The kinetics of PETCO_2_ decrease during the HVPT is partially dependent on plant gain. We previously demonstrated a decrease in plant gain in adults with alveolar hyperventilation (Bokov et al., 2016); thus, the absence of statistical significance for the group with alveolar hyperventilation may be related to the intermittent alveolar hyperventilation phenotype and the small sample size of the compared groups (*n* = 6). Our main result suggests that alveolar hyperventilation with CO_2_ depletion is encountered by all children with DB to some extent. This result further emphasizes that there is no relevant endotype of DB, at least based on hyperventilation and PCO_2_. Along this line, as previously stated, the importance of hypocapnia in inducing the associated symptoms of HVS has been questioned (Boulding et al., 2016).

We previously showed that the HVPT was unable to distinguish children with nonalveolar hyperventilation from children with DB without hyperventilation (Peiffer et al., 2022). We extend this result to the subtype of children with alveolar hyperventilation. The recovery phase of the HVPT was also non-significantly different for the children with physiological breathlessness on exercise and the children with DB (all subtypes). This result may seem unexpected, but the weak interest in the FETCO_2_ criteria of the recovery phase, which have not been validated, has been stated repeatedly (Vansteenkiste et al., 1991a). For instance, in the study of Vansteenkiste et al., neither the 3 min FETCO_2_ ratio nor the 5 min FETCO_2_ ratio during recovery after the HVPT showed a good correlation with the other “diagnostic” criteria (Vansteenkiste et al., 1991a; Vansteenkiste et al., 1991b) even if this study is often quoted as justifying these criteria (Tiotiu et al., 2021). Furthermore, from the study of Han et al. (1997) describing the linear recovery of FETCO_2_ during the HVPT, it can be calculated that healthy women recovered their baseline FETCO_2_ at ∼11 min (slope of increase = 0.0040%.s^−1^), while healthy men recovered their baseline FETCO_2_ at ∼10 min (slope of increase = 0.0045%.s^−1^). Thus, the usual and non-validated 5 min timepoint for defining delayed recovery should not be used (Pauwen et al., 2022).

The decrease in plant gain in the children with DB, whatever the subtype, implies that CO_2_ store depletion occurs in these children because of intermittent alveolar hyperventilation. It has been shown that hyperventilation results in an abrupt hyperbolic decrease in PaCO_2_ with a half-life time (time to reach ∼50% of its maximal change) of 3.2 ± 1.1 min (Giosa et al., 2021). The slow compartment stores of CO_2_ in the tissues is ∼90% of the total (Giosa et al., 2021). In the latter study, after the first hour, during which the CO_2_ store changes occurred primarily in the extracellular fluid, the changes in the different compartments were then almost linear, suggesting a similar loading or unloading rate from blood, interstitium, and the slow compartment stores. The decrease in plant gain traduces the unloading that may have occurred hours before the tests, which is a characteristic of DB, whatever the presence of hypocapnia during the tests. Furthermore, Giosa et al. (2021) showed that the extent of PaCO_2_ rise appeared to be a function of the previous CO_2_ store depletion. Thus, our results confirm a correlation between the slopes of decrease and increase in FETCO_2_. The decrease in loop gain at high frequencies (more stable ventilatory control) in children suffering from DB would attenuate the effects of the repetitive deep breaths on PaCO_2_ (hypocapnia) that could be evidenced in some patients with DB (Boulding et al., 2016).

From a clinical point of view, DB diagnosis does not require complex assessment such as plant gain measurement. When a child experiences resting dyspnea (88% in our series) that is not related to asthma exacerbation, she/he has allodyspnea that implies DB. When DB and asthma coexist, which is frequent, DB diagnosis is more complex (Sedeh et al., 2020; Tiotiu et al., 2021). In the presence of exertional dyspnea without resting dyspnea, the normality of the CPET with normal performance argues for physiological breathlessness if exertional dyspnea is related to sport practice. In the presence of limited performance during CPET, if an increase in V’E/V’CO_2_ slope, with or without hypocapnia, is evidenced, DB can be diagnosed. If limited performance is present without hyperventilation, poor muscular conditioning, which may generate dyspnea *per se*, (Plantier et al., 2014), can be discussed together with DB. Nevertheless, we previously showed that two standardized reassurance sessions afforded early clinical improvement and that a higher benefit in children with poorer muscular conditioning was observed (Mahut et al., 2014). Consequently, standardized reassurance sessions should be proposed for almost all children, with the exception of those with normal CPET performance and exertional breathlessness. Overall, the determination of DB subtypes would be useful if these subtypes are underpinned by different pathophysiological mechanisms, are stable over time and lead to different therapeutic approaches, which is not the case.

In conclusion, all children with DB, whatever their subtype, deplete their CO_2_ stores and thus experience a decrease in plant gain, which may be due to intermittent alveolar hyperventilation, suggesting the futility of their classification into subtypes. The HVPT describes CO_2_ kinetics related at least partially to plant gain.

## Data Availability

The raw data supporting the conclusion of this article will be made available by the authors, without undue reservation.
